# Monitoring fluoroquinolone resistance among ESBL-positive and ESBL-negative *Escherichia coli* strains isolated from urinary tract infections: An alert for empirical treatment

**DOI:** 10.1590/0037-8682-0513-2022

**Published:** 2023-04-14

**Authors:** Max Roberto Batista Araújo, Lincoln de Oliveira Sant’Anna, Nadir Nayara Carvalho dos Santos, Luisa Ferreira Seabra, Louisy Sanches dos Santos

**Affiliations:** 1 Instituto Hermes Pardini, Núcleo Técnico Operacional, Microbiologia, Belo Horizonte, MG, Brasil.; 2 Universidade do Estado do Rio de Janeiro, Departamento de Microbiologia, Imunologia e Parasitologia, Rio de Janeiro, RJ, Brasil.

**Keywords:** Fluoroquinolones, Urinary tract infection, Escherichia coli, Drug resistance

## Abstract

**Background:**

Bacterial resistance to extended-spectrum beta-lactamases (ESBL) is present worldwide. Empirical antibiotic therapy is often needed, and the use of fluoroquinolones, such as ciprofloxacin and norfloxacin, is common. This study aimed to analyze the urine cultures from 2,680 outpatients in January 2019, 2020, 2021, and 2022, with bacterial counts above 100,000 CFU/mL in which *Escherichia coli* was the etiological agent.

**Methods:**

We monitored the resistance of ESBL-positive and ESBL-negative strains to ciprofloxacin and norfloxacin and evaluated resistance rates.

**Results:**

Significantly higher fluoroquinolone resistance rates were observed among ESBL-positive strains in all years studied. Furthermore, a significant increase in the rate of fluoroquinolone resistance was observed between 2021 and 2022 in ESBL-positive and -negative strains, as well as from 2020 to 2021 among the ESBL-positive strains.

**Conclusions:**

The data obtained in the present study showed a tendency towards an increase in fluoroquinolone resistance among ESBL-positive and -negative *E. coli* strains isolated from urine cultures in Brazil. Since empirical antibiotic therapy with fluoroquinolones is commonly used to treat diverse types of infections, such as community-acquired urinary tract infections, this work highlights the need for continuous monitoring of fluoroquinolone resistance among *E. coli* strains circulating in the community, which can mitigate the frequency of therapeutic failures and development of widespread multidrug-resistant strains.

## INTRODUCTION

Urinary tract infections (UTIs) are among the most frequent infections in humans, with an incidence that is high in the community and varies with sex and age. UTIs can be caused by a range of pathogen, mainly *Escherichia coli* and other Gram-negative bacteria^1^. UTI treatment often requires the use of antimicrobials, among which oral beta-lactams and fluoroquinolones are the most prescribed until susceptibility results are acquired. However, owing to the rapid spread of drug resistance among Gram-negative microorganisms, including *E. coli*, UTIs are becoming increasingly difficult to treat^2^.

The production of extended-spectrum beta-lactamases (ESBL) is the most important resistance mechanism that Gram-negative bacteria have against beta-lactam agents^3^ ^-^ ^5^. Although there is no direct correlation between the mechanisms involved in resistance to beta-lactams and fluoroquinolones, ESBL-positive strains were found to be more resistant to these agents than ESBL-negative strains. Prior studies have demonstrated that until 58% of ESBL-positive strains harbor resistance genes against quinolones^6^ ^,^ ^7^.

Therefore, this study aimed to evaluate the frequency of resistance to ciprofloxacin and norfloxacin among ESBL-positive and ESBL-negative *E. coli* strains isolated from outpatients with UTIs in January 2019, 2020, 2021, and 2022.

## METHODS

In the present retrospective study, data were obtained from the database of one of the largest private laboratories in Latin America. Approximately 120,000 urine cultures originating from all regions of Brazil are processed monthly through an advanced automation system at the Operational Technical Nucleus, a central operation base located in the city of Vespasiano (Minas Gerais). The urine samples were collected at the service units spread throughout the country, preserved in boric acid (Greiner Bio-One^®^, Brazil), kept under refrigeration, and sent within 24 h to the central operation base. To ensure an adequate conservation time, the samples were transported by air through an integrated logistics system.

We randomly selected the results of 2,680 mid-stream urine cultures carried out in January 2019, 2020, 2021, and 2022 from the database. January was randomly chosen to standardize the time of year. Patients who answered the questionnaire with predefined information or were using antibiotics were excluded. To obtain an overview of the community strains, only information from outpatients was included in the study. Moreover, only data from cultures with bacterial growth above 100,000 CFU/mL, whose identification was conclusive for *E. coli* were included.

Until 2019, laboratory analyses were manually performed. The specimens were seeded in a laminar flow hood in chromogenic medium plates (chromID^®^ CPS^®^ Elite, bioMérieux^®^, Brazil) using a 1 μL calibrated loop. After incubation for 24 h at 37 °C in an aerobic atmosphere, biochemical tests were performed using a Modified Rugai medium (Renylab^®^, Brazil). Antimicrobial Susceptibility Tests (ASTs) were performed using the disk diffusion method in Mueller-Hinton medium (PlastLabor^®^, Brazil). Additionally, the strains were screened for ESBL production by the disk-approximation method using amoxicillin-clavulanic acid (20/10 µg, Oxoid^®^, Brazil), ceftriaxone (30 µg, Oxoid^®^), ceftazidime (30 µg, Oxoid^®^), aztreonam (30 µg, Oxoid^®^), and cefotaxime (30 µg, Oxoid^®^). Although several antibiotics were tested, the compiled results focused on the analysis of ciprofloxacin (5 µg, Oxoid^®^), norfloxacin (10 µg, Oxoid^®^), and ESBL production. Standardization followed Clinical and Laboratory Standards Institute (CLSI) guidelines^8^.

Since 2020, laboratory analyses have been conducted by using automated processes. The samples were processed using AutoPlack^®^ automatic seeders (Beckman Coulter Diagnostics^®^, USA) and bacterial identification was performed by MALDI-TOF mass spectrometry (VITEK^®^ MS, bioMérieux^®^, France). The ASTs and screening tests for ESBL were performed using semi-automated cards (VITEK^®^ XL, bioMérieux^®^). Standardization followed the guidelines established by the BrCAST^9^ and the Brazilian Ministry of Health. The tests were quality controlled using the standard strains *E. coli* ATCC 25922 (ESBL-negative), *Pseudomonas aeruginosa* ATCC 27853 (ESBL-negative), and *Klebsiella pneumoniae* ATCC 700603 (ESBL-positive).

The fluoroquinolone resistance rates among ESBL-positive and -negative groups were calculated and analyzed using the OpenEpi software (version 3.0.1) (Dean AG, Sullivan KM, Soe MM. OpenEpi: Open Source Epidemiologic Statistics for Public Health, http://www.OpenEpi.com). A Chi-square test was performed to compare the resistance rates between the studied groups.

## RESULTS

Resistance frequency to ciprofloxacin and norfloxacin was evaluated in 1,340 ESBL-positive (335 per year) and 1,340 ESBL-negative (335 per year) *E. coli* strains randomly selected during the same period (January) of 2019, 2020, 2021, and 2022. Approximately 43.2% (*n*=1,158) of the *E. coli* strains were found to be susceptible to the tested fluoroquinolones, whereas the rest, 56.8% (n=1,522), showed resistance to both antimicrobials.

**FIGURE 1:**
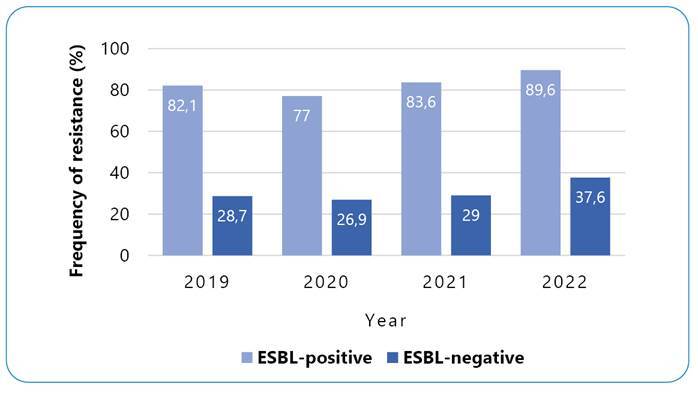
Fluoroquinolone resistance among extended-spectrum beta-lactamases (ESBL)-positive and -negative *Escherichia coli.* Frequency of resistance in each of the groups analyzed individually, demonstrating the trend over the years of study.

As shown in Table 1 and Figure 1, the frequency of fluoroquinolone resistance in the ESBL-negative strains was 28.7% (n=96) in 2019, 26.9% (n=90) in 2020, 29% (n=97) in 2021, and 37.6% (n=126) in 2022. The resistance of ESBL-positive strains was verified to be 82.1% (n=275) in 2019, 77% (n=258) in 2020, 83.6% (n=280) in 2021, and 89.6% (n=300) in 2022.

The fluoroquinolone resistance rates among ESBL-positive strains compared to ESBL-negative strains were significantly different (*p* < 0.0001) in each of the years considered individually. However, there was no statistically significant difference in resistance when the years were compared, specifically, 2019 versus 2020 for ESBL-positive isolates, and for ESBL-negative isolates, in 2019 versus 2020 and 2020 versus 2021. In all other evaluations between years, we found a statistically significant increase in fluoroquinolone resistance as shown in Table 1.

**TABLE 1:** Frequency of resistance/susceptibility to ciprofloxacin and norfloxacin among extended-spectrum beta-lactamases (ESBL)-positive and -negative *Escherichia coli* observed in January 2019, 2020, 2021, and 2022.

		Years														
Group (n)	Fluoroquinolone profile (n)	2019			2020			2021			2022			***p* value 2019-2020**	***p* value 2020-2021**	***p* value 2021-2022**
		n	%	***p* value**	n	%	***p* value**	n	%	p value	n	%	***p* value**			
ESBL-positive (1340)	Resistant (1113)	275	82.1	<0.001	258	77.0	<0.001	280	83.6	<0.001	300	89.6	<0.001	0.051	0.016	0.023
	Susceptible (227)	60	17.9		77	23.0		55	16.4		35	10.4				
ESBL-negative (1340)	Resistant (409)	96	28.7		90	26.9		97	29.0		126	37.6		0.302	0.363	0.017
	Susceptible (931)	239	71.3		245	73.1		238	71.0		209	62.4				

**Notes**: In all analyses, the difference between ciprofloxacin and norfloxacin resistance was below 1%. For a better understanding, we considered them equal and named them fluoroquinolone resistant. p value < 0.05 was considered statistically significant.

## DISCUSSION

In routine laboratory tests, several microorganisms can be isolated from urine cultures. Enterobacteria are the main etiological agents of UTIs with *E. coli* being the most frequent species. The widespread use of empirical antibiotic therapy significantly contributes to the increased prevalence of antimicrobial-resistant strains, as the indiscriminate and incorrect use of therapeutic agents is a risk factor for the emergence and spread of microbial resistance^6^ ^,^ ^10^ ^,^ ^11^.

Resistance to beta-lactams is often reported for Gram-negative bacteria, occurring mainly due to the production of beta-lactamases or associated with changes in membrane permeability due to the loss of porins. Resistance depends on the amount of enzyme present and the affinity for the substrate, and may not be measured by AST, if the suspension of microorganisms is not adequate and variation in the predominant enzyme can also occur. Therefore, routine susceptibility tests do not always detect ESBL production, but the possibility of their presence cannot be ignored and is relevant^12^ ^-^ ^14^.

The treatment of infections caused by resistant strains offers a substantial challenge, as they can hydrolyze penicillin, cephalosporins of all generations, and monobactam, minimizing therapeutic options, and only some beta-lactam antibiotics maintain their activity against them^15^. To aggravate this issue, two additional facts must be highlighted. First, empirically prescribed antimicrobials that reach a percentage of resistance above 20% in the community run a high risk of therapeutic failure, recommending caution with their usage. Second, a significant increase in the appearance of ESBL-producing strains has been reported worldwide, including in North America and South/Latin America^16^ ^-^ ^20^.

Although fluoroquinolone resistance in *E. coli* is not new, studies investigating the resistance rates among strains circulating in the community are necessary, especially given the spread of multidrug-resistant (MDR) strains and the recurrent use of empirical therapy with these antimicrobials for the treatment of UTIs. Thus, in the present study, we analyzed the frequency of fluoroquinolone resistance among ESBL-positive and ESBL-negative *E. coli* strains isolated from outpatients with UTIs in the same month over the past four years.

Our data revealed that the rate of fluoroquinolone resistance in the ESBL-positive strains was approximately 180% higher than that in the ESBL-negative samples in all years evaluated. In addition, our data showed a trend towards increased rates of fluoroquinolone resistance among both ESBL-positive and -negative strains. Once ESBL-negative strains are isolated at high levels in a community, these findings can be considered an alert for the empirical treatment of UTIs.

In 2020, the microbiology laboratory standards were changed. A committee formed by members of the Societies of Clinical Analysis, Infectious Diseases, Microbiology, Clinical Pathology, and Laboratory Medicine began to determine and periodically review procedures for the interpretation of susceptibility tests to antimicrobials for clinical use and epidemiological purposes, proposing to the National Agency of Sanitary Surveillance the implementation of these procedures in Brazilian clinical laboratories. The CLSI standards fell into disuse, and this change was implemented in our study at its inception. Thus, it is necessary to consider the change in breakpoints for fluoroquinolones. For example, resistance to ciprofloxacin was previously determined by a minimum inhibitory concentration of ≥1 µg/mL but is now determined when >0.5 µg/mL. Initially, we believed that this would increase the number of resistant strains. However, as the change was only one dilution point, this was not occurred. In fact, as shown in Table 1, the number of resistant strains in 2020 was not statistically different from that in 2019, with *p* values of 0.051 and 0.302 (resistance in ESBL-positive and ESBL-negative, respectively). For the ESBL-negative group there was a decrease in the absolute number (from 96 to 90 strains).

In Brazil, a similar study found a fluoroquinolone resistance rate of approximately 76%^21^ among ESBL-positive strains. In Latin America, resistance rates of 58% and 90% have been reported in Peru^22^ and Venezuela^23^, respectively. Moreover, a rate of 80% has been reported in Mexico, 60% in Ecuador^24^ and 12% in Chile^25^. In Brazil, rates of 43%^26^ and 56%^27^ have been reported. In earlier studies, rates of 12%^28^ ^,^ ^29^ and 22%^30^ were found. In a Russian study, a resistance rate of 25% was obtained in 2017, which was 23% higher than the rate found in 1999^31^.

Studies have also indicated that microorganisms with the ESBL phenotype may show *in vitro* susceptibility to some drugs, but the use of these drugs leads to a lower clinical response due to the inoculum effect, resulting in an increase in the minimum inhibitory concentration when faced with a large bacterial inoculum or when the drug fails to reach its pharmacodynamic targets^32^ ^,^ ^33^. Thus, the decrease in antimicrobial susceptibility of ESBL-positive strains may also represent a significant possibility for therapeutic failure in situations where AST is not performed.

In cases of UTIs, particularly community-acquired UTIs, it is common to not perform urine cultures and apply empirical treatment. However, with the high rates of resistance in ESBL-positive strains, empiric therapy can result not only in therapeutic failure, but also increase bacterial resistance rates, which is an unfavorable outcome. Infection control is extremely important, and studies such as this one have value in guiding health professionals in this difficult mission. Bacterial multidrug resistance must be considered, and therapeutic options, which are often limited, become even scarcer when ESBL-producing bacteria cause infection.

The clinical significance of ESBL strains is high because limitation in antibiotic choice for treatment, thus making it evident that monitoring the resistance profile of bacteria is beneficial and recommended, as it reduces the chance of failure and spread of MDR strains. Finally, increased fluoroquinolone resistance among ESBL-negative strains, which is more common in the community (non-hospital environments), should also be monitored to prevent rising resistance rates in infections.

## ETHICAL APPROVAL

Since the study was a retrospective analysis of laboratory data collected, and no additional investigations were performed with submitted specimens, no ethical approval was applied.
